# A Look Into the Cytotoxicity of Composite Fillings: Friend or Foe?

**DOI:** 10.7759/cureus.46327

**Published:** 2023-10-01

**Authors:** Sayem A Mulla, Saba A Kondkari, Amit Patil, Ashish Jain, Sheetal Mali, Himmat C Jaiswal, Ashima Jakhar, Zoha M Ansari, Sumeet Agarwal, Pooja Yadav

**Affiliations:** 1 Dentistry, Bharati Vidyapeeth (Deemed to be University) Dental College and Hospital, Navi Mumbai, IND; 2 Conservative Dentistry and Endodontics, Bharati Vidyapeeth (Deemed to be University) Dental College and Hospital, Navi Mumbai, IND; 3 Prosthodontics, Bharati Vidyapeeth (Deemed to be University) Dental College and Hospital, Navi Mumbai, IND

**Keywords:** restorative dentistry, dental composite, bisphenol a-glycidyl methacrylate, mtt assay, monomer, cytotoxicity, composite, bis-gma

## Abstract

Dental resin composites are widely used restorative materials in dentistry for the treatment of carious and non-carious lesions as well as pit and fissure sealants, cavity liners, and endodontic sealers. They consist of two parts: an organic resin matrix and an inorganic/organic filler. The organic resin matrix phase is made up of multifunctional monomers and light-sensitive initiators, while the inorganic/organic filler phase is made up of micro/nano-sized fillers that primarily serve as reinforcement. Despite being a very promising dental material, its monomeric component has some drawbacks. It is well known for leaching out during incomplete polymerization, which can result in cytotoxicity. Bis-GMA (bisphenol A-glycidyl methacrylate) is the most cytotoxic of all monomeric components that exhibit synthetic estrogenic effects. The purpose of this article is to assess the cytotoxic effects of dental composite, understand the possible mechanism behind them, and explore ways to screen for and reduce this harmful effect, as well as shed light on its future prospects.

## Introduction and background

Restorative dentistry is a pertinent branch of dentistry that entails the restoration of defective teeth preferably with tooth-mimicking materials. For a long time, a number of materials like amalgam, gold, etc. have been used in the restoration of teeth. The dental material that is currently being used by most dentists in restorative dentistry is named "composite". This tooth-colored material is popular for giving the teeth a natural appearance and is thus well-known and in demand for various aesthetic procedures. However, because of its monomeric component, which is notorious for being cytotoxic in nature, the biocompatibility of this material is an important issue that demands special attention [1].

Organic resin matrix, particles of inorganic fillers, silane coupling agent, and initiators/accelerators and pigments make up the resin composite [2]. High viscosity along with multiple functional groups of bisphenol A-glycidyl methacrylate (Bis-GMA) and urethane dimethacrylate (UDMA) are all indications of a low viscosity monomer like triethylene glycol dimethacrylate (TEGDMA) as the diluent monomer [3].

The risks associated with insufficient polymerization, i.e., creating a biological reaction, have been specified for all the materials used in restorative dentistry including those that can be polymerized [4]. Although complete polymerization of dental composites is theoretically achievable, it is a well-known fact that methacrylate monomers (MMA) are composed of double-bonded carbon-carbon (C-C) groups, and almost 25-50% of MMA often remain unreactive after the polymerization. Studies have reported that these monomers, which were not completely polymerized, traveled through the dentinal tubules and reached the dental pulp, leading to biological reactions [5].

Immersion of resin composites in fluid mediums such as saliva or water, the residual uncured monomers can leach out and cause local or systemic effects [6]. Several in vitro studies have been conducted to determine the toxicity of methacrylate monomers. Despite the fact that the precise mechanism behind this toxic effect is yet to be determined, a few researchers have found that the decrease in the levels of cellular glutathione (GSH) [7] and higher levels of reactive oxygen species (ROS) [5] represents the initial events in a plethora of cell types. Higher levels of ROS have been proposed as the prime cause of damage to the DNA in cells exposed to methacrylate [8].

As materials such as TEGDMA and BisGMA are in close proximity to the dental pulp, it is critical to understand their biocompatibility and cytotoxic effect. The purpose of this review is to highlight the cytotoxic effects of composites on the human body, understand the possible mechanisms underlying this cytotoxicity, explain the tests used to diagnose cytotoxicity, and shed light on their future prospects.

## Review

What is cytotoxicity?

Cytotoxicity is defined as the toxicity caused by certain substances in living cells [9]. Biocompatibility has been defined as a living system's reaction to exogenous materials [10]. These two terms are synonymous. To have the least cytotoxic effects, any dental material placed in the oral cavity, whether near the gingiva or the pulp, should be biocompatible.

Cytotoxic effects

According to studies, there are various harmful effects of composites (Table 1) [11-17], although conventional composites have lower cytotoxic and genotoxic effects in comparison to uncured forms. These uncured forms of composites exhibit a high degree of cytotoxicity and genotoxicity due to their monomeric components which cause oxidative stress [18].

**Table 1:** Various toxic effects associated with dental composites.

Hazards	Effects	Reference
Endocrinal disturbances	Impaired hormone productions	[11]
	Type 2 diabetes (impacts insulin resistance)	
	Weight gain	
Reproductive disorders	Impaired egg maturation	[12]
	Infertility defined as “lifelong and transgenerational” in women	
	Erectile dysfunction and libido issues in males	[13]
Heart diseases	Coronary artery diseases	[14]
	Angina	
	Heart attack	
	Hypertension	
	Arrythmias	
	Atherosclerosis	
Fetus	Impacts brain development	[15]
	Anxiety issues	
Cancer	Breast cancer in females	[16]
	Prostate cancer in males	
Respiratory system	Asthma	[17]

In a study by Nascimento et al., two conventional resins, nine bulk-fill resins, and 11 different types of composite resins were tested on L929 fibroblast cell lines for cytotoxicity using the MTT (3-(4,5-dimethylthiazol-2-yl)-2,5 diphenyl tetrazolium bromide) assay and the neutral red uptake assay. The results revealed that the mentioned materials had minimal or no cytotoxic effects when compared with the control group [19].

The effect of preheating on the cytotoxicity of bulk-filled composite resins on human fibroblast cells was investigated by Chaharoom et al. [20]. Three different types of composite resins were studied, and the results showed no difference in cytotoxicity in terms of preheating.

In a study by Dreanca et al., graphene dental composite was subjected to cytotoxicity tests on human dental follicle stem cells and showed no toxic effects against stem cells after 24 hours [21]. In vivo studies on rats were also done; seven week later, the bone was harvested and subjected to histological testing, which reported the materials' lack of systemic organ toxicity.

A study targeted to assess the cellular response to biphasic calcium phosphate (BCP) ceramics was done using in vitro assessment of cytotoxicity via fibroblast cells of mouse. BCP, composed of β-tricalcium phosphate (β-TCP) and hydroxyapatite (HAp), was created by stirring solutions of propagated coral and dicalcium phosphate anhydrous, which was then subjected to heat treatments at 1100 °C for one hour to seven days. The synthesized BCP samples which tested negative for cytotoxic effects were further implanted in the rabbit model’s bone defects. This did not lead to any local adverse cellular or tissue reactions and instead depicted new bone formation and growth [22].

A study by Camassa et al. showed that dental composite dust can induce a toxicity response on human bronchial epithelial cells HBEC-3KT in vitro [23]. The results showed that the toxic effect develops at high doses and after more than 24 hours of exposure. Moreover, dust generated by a super coarse diamond bur appears to affect the integrity and cell viability more than dust from a fine diamond bur. This knowledge is especially crucial for dentists in order to sustain a healthy lifestyle.

A study was conducted by Kavuncu et al. to determine the cytotoxicity of various nano-composite resins on human periodontal ligament fibroblast cells (hPDLF) and human gingival fibroblast cells (hGF) [24]. The results of an MTT assay after eight days revealed that CT was more cytotoxic to hGF cells than the control. When it comes to restorative cases involving gingiva and PDL, the nano-hybrid ormocer composites are more biocompatible because AF did not exhibit cytotoxicity to either of the cell lines.

In another such study, eight different types of monomers were used and their cytotoxicity was tested on immortalized hGF-1 cell lines [25]. Cell viability was determined using an MTT assay, and the results suggested that different composites had different biocompatibility standards based on the number of unbound monomers present in them. Fewer monomers were released by nano-hybrid ormocers and showed less cytotoxic effects. It also revealed that ceramic fillers-based composites were more biocompatible.

Gupta et al. found that the cytotoxicity of composites of the deeper shade (C2) had higher cytotoxicity. Their study showed that quick curing with a high-power unit would be advantageous for composites in terms of reducing the release of harmful chemicals [26]. 

A comparative in vivo study of the evaluation of cytotoxicity and genotoxicity of composites in human gingival cells suggested that the highest cytotoxic effects were shown by flowable composites followed by bulk-fill composites [27]. There was no genotoxicity induced by any materials. Furthermore, there was no evidence of long-term ill effects of composites.

After different incubation periods in artificial saliva, the cytotoxicity of two indirect composite resins was tested on fibroblast cells [28]. The cytotoxicity of two indirect composite resins was evaluated on fibroblasts. The results revealed that both test materials were moderately toxic to the cells. The researchers concluded that soaking the samples in artificial saliva for 48 hours had a significant effect on cytotoxicity, recommending that clinicians soak the resin prosthesis in water or artificial saliva for at least 48 hours before placing it in the patient's mouth.

In a study to test whether the curing system and light units have an effect on cytotoxicity, it was concluded that dual curing systems were more biocompatible than light curing systems [29].

Composites are believed to be less toxic than silver-reinforced glass ionomer cement, Giomer, conventional glass ionomer cement, and resin-modified glass ionomer cement [30].

Possible mechanism of dental composite cytotoxicity

**Figure 1:**
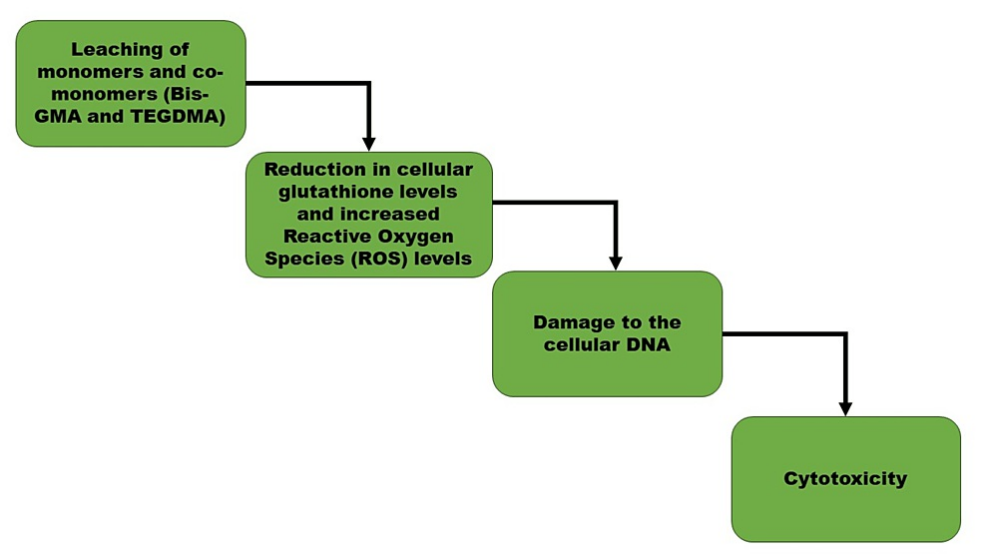
Possible mechanism behind dental composite cytotoxicity Bis-GMA: bisphenol A-glycidyl methacrylate; TEGDMA: triethylene glycol dimethacrylate

The possible mechanism (Figure 1) behind the cytotoxicity of dental composites, which can lead to local adverse effects (pulpal changes, marginal gingivitis, and allergic reactions) along with different systemic effects and allergies [31], cytotoxicity [32], genotoxicity, and even toxic effects on the reproductive system [33], is complex. The precise mechanism by which this occurs is yet to be determined in the literature; however, the reduction in the levels of cellular glutathione [34] and increased ROS levels are believed to be the major triggering factors [35]. Furthermore, the latter has been linked to DNA damage in cells exposed to methacrylate monomers [8].

Another possible mechanism is the release of bisphenol A (BPA) from BPA-based monomers such as BIS-GMA, which acts as an endocrine disruptor [36]. BPA can act as a xenoestrogen making it an endocrine-disrupting chemical (EDC). EDCs can act as hormone mimics, interfere with natural hormone binding to receptors, or both. As a result, they amplify the effects of endogenous hormones [37].

Tests used to evaluate cytotoxicity

Cytotoxicity tests are widely used in vitro for a variety of reasons, including convenience, reproducibility, and avoidance of animal suffering. However, the testing procedures of some traditional in vitro cytotoxicity tests, such as agar or filter diffusion tests or extract tests, do not correspond with clinical practice [38]. All of the tests conducted to date to assess the cytotoxicity of dental materials have yielded positive results. In the case of dental composites, unpolymerized monomers such as Bis-GMA, UDMA, HEMA (2-hydroxyethyl methacrylate), TEGDMA, camphorquinone, and glutaraldehyde are primarily responsible for the cytotoxic effects [39-43].

MTT Assay

For a cell to be viable, its mitochondrial activity needs to be constant. Therefore, an increase or decrease in the number of viable cells is directly proportional to its mitochondrial activity. MTT assay is based on the principle of conversion of tetrazolium salt MTT to formazan crystals. These formazan crystals determine the mitochondrial activity of the cell. Hence, an increase or decrease in the viable cell count can be determined by measuring the formazan concentration [44].

Neutral Red Uptake Assay

A cell viability assay that enables in vitro assessment of xenobiotic-induced cytotoxicity is the neutral red uptake assay. The assay depends on living cells' capacity to bind and integrate neutral red, a weak cationic dye, in lysosomes. A concentration-dependent reduction of the uptake of neutral red after exposure to the xenobiotic under investigation confirms the cytotoxicity [45].

Flow Cytometry

Flow cytometry is used to evaluate the cell cycle and cell death. The cultured cells; usually pulpal or gingival fibroblasts in the case of the oral cavity, are subjected to propidium iodide (PI) staining and the graph is plotted [46-48].

Quantitative Real-Time Polymerase Chain Reaction (RT-PCR)

To perform this particular cytotoxicity test, RNA from cells is extracted using an RNX-plus extraction kit, followed by cDNA synthesis completion and subsequent RT-PCR [49].

Agar Diffusion Test

The cytotoxicity of elastomeric closures is evaluated using agar diffusion tests. The agar layer in this test behaves like a cushion. Agar has a role in protecting the cellular component from a myriad of mechanical damages while also allowing any sort of leachable chemical entities to diffuse from the packaging samples or the product. The cells obtained are then tested to see if the collected samples are toxic. Extracts from the material are applied to a piece of filter paper, and then the cytotoxicity analysis can be done using the agar diffusion test. Cultures are incubated at 37.1° C in a humidified incubator containing 5% CO2. After incubation, cells exposed to samples, positive controls, and negative controls are stained or evaluated under a microscope without staining. The biological reactivity of cells exposed to the sample or sample extracts is rated on a scale of 0-4. Nonlethal injury (cellular degeneration) and any structural defects (malformations) are used to establish the biological reactivity of the cells. The agar diffusion test is considered valid if grade 0 is observed in the responses to the negative controls while the positive controls at least attain grade 3. The requirements of the agar diffusion test are met if the samples’ biological response is not greater than grade 2 [50].

Confocal Microscopy

Confocal imaging's primary goal is to create a three-dimensional (3D) architecture of cells and organs. The technique can reveal not only the surface of a specimen but also its subsurface. The protocol describes the use of 3D confocal laser scanning microscopy (CLSM) time-lapse imaging as a sensitive method for assessing a dental composite's in vitro biocompatibility behavior. Using live or dead staining, in vitro evaluation is performed on cultured primary hGF cells. After that, the images (FV10i confocal biological inverted system) are obtained, studied, and analyzed using software such as FV10-ASW 3.1 software (Olympus Life Science, Tokyo, Japan) [51].

Handling dental composite cytotoxicity in clinical practice

Greater cytotoxicity is produced by shorter curing times at higher light intensities emitted by the curing light source than by longer curing times at lower intensities [51]. The addition of ceramic fillers has been shown to reduce the amount of monomer release and, as a result, can be considered for use in the composite composition for application in the oral cavity. However, more research is needed [25]. Due to the lack of cytotoxicity of Admira Fusion (VOCO GmbH, Cuxhaven, Germany) on hGF and hPDLF cells, ormocer composites can be considered as a biocompatible alternative in clinical situations where a restoration in close association with the gingiva and periodontal ligament is required. It is possible to draw the conclusion that the supra-nano-hybrid resin-based composite material Estelite Quick Sigma (EQS) (Tokuyama Dental Corporation, Tokyo, Japan), which contains Bis-GMA and TEGDMA, is not cytotoxic to hGF and hPDLF cells. This is most likely a result of decreased monomer releases due to the improved polymerization technology. Further research into the effects of a new TCD-DI-HEA (tricyclodecane dimethanol diacrylate hexafluorophosphate) monomer, which contains Charisma® Topaz (Kulzer GmbH, Hanau, Germany) on the human periodontium cells is necessary due to its cytotoxic effects on hGF and hPDLF cells [24]. Removal of the oxygen-inhibition layer results in the least quantity of cytotoxicity. To improve the biocompatibility of dental composites, clinicians should immediately remove the unreacted monomer particles from the resin surface after restoring the tooth with light-curing resin [49].

Limitations and future prospects

The major drawback or limitation of the current literature on dental composite cytotoxicity is the lack of in vivo studies to test cytotoxicity. There is a need for more clinical studies for cytotoxicity testing. Furthermore, there is a need for research in newer dental composite materials or newer clinical techniques with low or no toxicity to humans. 

## Conclusions

Even though dental composites hold an added advantage due to their close resemblance to the tooth in terms of color, there is still scope for betterment in regard to their composition and clinical usage technique. The addition of different fillers like ceramic and proper curing along with a reduction in monomer content can decrease cytotoxic effects associated with dental composites. More in-depth in vivo studies and clinical systematic reviews can pave the way for much more biocompatible dental composites in dentistry.
